# Leuconostoc sp. Meningitis in a Patient Treated with Rituximab for Mantle Cell Lymphoma

**DOI:** 10.4274/tjh.2013.0149

**Published:** 2015-08-01

**Authors:** Hrvoje Holik, Božena Coha, Marijan Šiško, Maja Tomić-Paradžik

**Affiliations:** 1 General Hospital Dr. Josip Benčević, Clinic of Internal Medicine, Slavonski Brod, Croatia; 2 General Hospital Dr. Josip Benčević, Clinic of Infectious Diseases, Slavonski Brod, Croatia; 3 Public Health Institute of the Brod-Posavina County, Department of Microbiology, Slavonski Brod, Croatia

**Keywords:** Rituximab, Leuconostoc, Purulent meninigitis, mantle cell lymphoma, R-CHOP

## Abstract

We present a 64-year-old man who was treated with R-CHOP (rituximab plus cyclophosphamide, doxorubicin, vincristine, and prednisolone) chemoimmunotherapy for mantle cell lymphoma and developed purulent meningitis, probably caused by Leuconostoc sp. The patient had severe hypogammaglobulinemia, which is a possible complication of rituximab therapy. To our knowledge and after reviewing the available medical literature, this is the first described case of purulent meningitis caused by Leuconostoc sp. in a patient with mantle cell lymphoma that appeared after treatment with the R-CHOP protocol. The diagnosis of purulent meningitis was based on clinical, laboratory and cytological cerebrospinal fluid findings, in addition to blood culture results in which we isolated Leuconostoc sp. The patient was treated with meropenem with full recovery.

## Abstract (tr)

Burada mantle hücreli lenfoma tanısı ile R-CHOP (rituksimab, siklofosfamid, doksorubisin, vinkristin, prednizolon) kemoimmünoterapisi ile tedavi edilen ve muhtemelen Leuconostoc cinsi etkene bağlı pürülan menenjit gelişen 64 yaşında bir erkek hastayı sunuyoruz. Hastanın rituksimab tedavisinin olası komplikasyonlarından biri olan ağır hipogammaglobulinemisi bulunmakta olup, bildiğimiz kadarı ile ve mevcut tıbbi literatürün taranması sonrasında, olgumuz R-CHOP protokolü ile tedavi sonrası Leuconostoc cinsi etkene bağlı pürülan menejit gelişen mantle hücreli lenfoma tanılı ilk hastadır. Pürülan menenjit tanısı klinik bulgular, laboratuvar ve beyin-omurilik sıvısının sitolojik bulguları ve Leuconostoc izole ettiğimiz kan kültürünü temel alıyordu. Hasta meropenem tedavisi ile tamamen iyileşti.

## INTRODUCTION

Rituximab is a chimeric monoclonal antibody that binds to the CD20 cell surface marker on B lymphocytes. It was approved in the United States in 1997 by the Food and Drug Administration and now is used in 3 major fields: hematological oncology, prevention of transplant rejection, and some autoimmune diseases [1]. After 15 years of global use, due to expansion of its indications and duration of therapy, we begin to see side effects of rituximab therapy that were not previously known [1].

The genus Leuconostoc comprises microorganisms previously classified as Lancefield group N streptococci. They are facultative anaerobic, catalase-negative, gram-positive, vancomycin-resistant organisms of cocci form [2]. In nature they are often found on the surfaces of plants, vegetables, and dairy products. They are important bacteria in today’s industry, especially in food fermentation [3]. Leuconostoc sp. mainly cause opportunistic infections in immunocompromised patients [3].

## CASE PRESENTATION

A 64-year-old man was admitted to our hospital in March 2011 with generalized lymphadenopathy and hepatosplenomegaly. Based on the clinical and histopathological findings of a resected neck lymph node and epipharyngeal tumor, we diagnosed mantle cell lymphoma stage IV B. The patient was treated with the R-CHOP (rituximab plus cyclophosphamide, doxorubicin, vincristine, and prednisolone) protocol and received 6 cycles of chemoimmunotherapy in 3-week intervals with very good response. He achieved complete remission.

Serum protein electrophoresis performed at diagnosis on 25 March 2011 revealed the following: albumin: 35.58 g/L, alpha 1: 2.94 g/L, alpha 2: 6.3 g/L, beta: 7.74 g/L, gamma: 7.44 g/L. Forty days after the last application of chemoimmunotherapy, on 23 September 2011, he was admitted to the department of infectious diseases of our hospital. At admission he was in a poor condition with severe headache, fever of up to 38.8 °C, and double vision. Upon clinical examination we found that he was conscious and dehydrated, with neck stiffness. Based on these clinical findings, cerebrospinal fluid findings (cells: 3408/3x106/L, erythrocytes: 796x106/L, glucose: 0.9 mmol/L, Cl: 99 mmol/L, protein: 1.04 g/L, 0.87 Sayk neutrophil granulocytes, lymphocytes: 0.10, monocyte-macrophageal cells: 0.03), and the blood culture findings in which Leuconostoc sp. was isolated, he was diagnosed to have purulent meningitis. Parameningeal focus such as sinusitis, mastoiditis, otitis media, brain abscess, or spinal epidural abscess was ruled out. The patient lived in the countryside with constant contact with dairy products and other potential Leuconostoc habitats, so he had the opportunity to be infected with Leuconostoc. In the blood sample results at admission there were 10.1x109/L leukocytes with 83% neutrophils, the C-reactive protein level was 131 mg/L, and the lactate dehydrogenase level was 117 U/L. Electrophoresis of serum proteins showed severe hypogammaglobulinemia (30/09/2011: albumin: 34.95 g/L, alpha 1: 1.15 g/L, alpha 2: 2.1 g/L, beta: 8.4 g/L, gamma: 3.4 g/L). Sensitivity of the isolated pathogen as tested by the Kirby-Bauer method of disk diffusion and the minimum inhibitory concentration (MIC) value for each antibiotic was determined by the E-test method. The pathogen was sensitive to all beta-lactam antibiotics (penicillin MIC of 0.064 mg/L), imipenem (MIC of 0.50 mg/L), macrolides, lincosamides (clindamycin MIC of 0.125 mg/L), and quinolones but was resistant to vancomycin (MIC >256 mg/L). The patient was initially treated with ceftriaxone (2x2 g intravenous), then with ampicillin (6x2 g intravenous) and gentamicin (1x240 mg intravenous), and finally with meropenem (3x2 g intravenous), according to the blood culture findings. With that therapy, his general condition improved with normalization of laboratory parameters of inflammation and he no longer had fever. Control electrophoresis of serum proteins performed before discharge showed an increase in gamma-globulin levels (serum protein electrophoresis, 14/10/2011: albumin: 46.53 g/L, alpha 1: 0.96 g/L, alpha 2: 3.84 g/L, beta: 7.04 g/L, gamma: 5.63 g/L). At the time when patient was treated our biochemistry laboratory could not performed fractions of gamma globulins, so we just have total value. The patient was discharged in good condition with full recovery.

## DISCUSSION AND REVIEW OF THE LITERATURE

To our knowledge and after reviewing the available medical literature, we conclude that this is the first described case of purulent meningitis that was probably caused by Leuconostoc sp. in a patient with mantle cell lymphoma. Meningitis appeared after treatment with the R-CHOP protocol. For certain diagnosis of purulent meningitis caused by Leuconostoc, we lack cerebrospinal fluid culture with isolated Leuconostoc. Antibiotics having been administered before the lumbar puncture could be the reason why the cerebrospinal fluid culture was negative. The factors in favor for infection with Leuconostoc were isolation of Leuconostoc from blood culture, the patient being immunocompromised, and good and quick response to the prescribed antibiotic therapy with full recovery. In the literature, most descriptions of infections with Leuconostoc are in neonatal and other pediatric patients [4,5,6,7,8,9]. Infections with Leuconostoc in adults are rare. Leuconostoc can cause endocarditis, urinary tract infections, meningitis, intraabdominal infections, bacteremia, and septicemia. Central venous catheters and total parenteral nutrition are risk factors for infections with Leuconostoc, while newer findings showed that previous antibiotic treatment with vancomycin is not a risk factor for Leuconostoc infections [3,7,10]. In most cases, Leuconostoc showed good sensitivity to carbapenems (imipenem, meropenem), gentamicin, tobramycin, chloramphenicol, and the more recent daptomycin [3]. However, there is also a report of Leuconostoc resistance to carbapenem antibiotics [11].

In the literature, bacterial or viral infections associated with the use of rituximab were described in patients who were receiving maintenance therapy [1,12,13]. In randomized trials in which rituximab was not administered as maintenance therapy, there was no increase in the incidence of infections in patients who were treated with the addition of rituximab to chemotherapy as compared to chemotherapy alone [1,14]. The most common infections described in the literature are reactivation of hepatitis B virus, progressive multifocal leukoencephalopathy caused with JC polyomavirus, enterovirus encephalitis, parvovirus B19 infection, Pneumocystis jirovecii pneumonia, babesiosis, infections with West Nile virus, and cytomegalovirus [1,15]. Some of the bacterial infections associated with the use of rituximab described in the literature are Staphylococcus warneri meningitis with Strongyloides stercoralis superinfection in a patient with mantle cell lymphoma and tracheobronchitis caused by Pasteurella multocida in a patient with chronic lymphocytic leukemia [16,17]. Both patients were treated with rituximab as maintenance therapy. In patients with non-Hodgkin’s lymphoma who were treated with rituximab as a maintenance therapy and who had developed different degrees of hypogammaglobulinemia, staphylococci and Escherichia coli were reported as causes of bacterial infections [13]. In another study, 20% of patients treated with rituximab and chemotherapy developed infections without neutropenia. In all cases in which electrophoresis of serum proteins was performed, different degrees of hypogammaglobulinemia were observed. These patients were successfully treated with intravenous gamma globulins [18].

Here we have presented a patient who developed severe hypogammaglobulinemia after only 6 cycles of rituximab, for whom infection occurred 40 days after the last cycle. Besides application of rituximab, the potential risk factors were underlying lymphoproliferative disease and immunosuppressive therapy usage. Although rituximab is a safe drug for use, further research is necessary about its use in certain groups of patients who might develop an opportunistic infection. Controlling the value of gamma globulins during rituximab therapy might reduce the number of infections by temporary interruption of rituximab or by giving prophylactic intravenous gamma globulins in cases of severe hypogammaglobulinemia. Expansion of indications for rituximab use and increasing duration of treatment with rituximab will certainly bring about more opportunistic infections, such as those caused by Leuconostoc.
